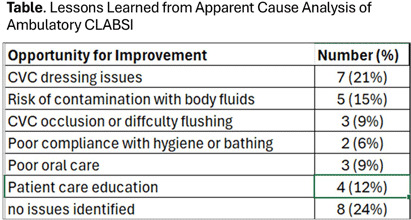# Apparent cause analysis identifies opportunities for improvement to reduce ambulatory central line associated bloodstream infections

**DOI:** 10.1017/ash.2025.344

**Published:** 2025-09-24

**Authors:** Sarah Carmack, Megan Key, Julie Morganelli, Shilpa Gorantla, Rafonda Burks, Craig Gilliam, Hana Hakim

**Affiliations:** 1 St. Jude Children’s Research Hospital

## Abstract

**Background:** Pediatric oncology patients receive significant care in outpatient settings using central venous catheters (CVC) for chemotherapy, blood product transfusions, parenteral nutrition, and blood tests. Data about the rate of ambulatory central line associated bloodstream infection (CLABSI) is limited and its prevention activities have been derived from the inpatient setting with potentially limited applicability to the outpatient care. In this quality improvement project, we used apparent cause analysis (ACA) to better identify barriers and facilitators to reduce ambulatory CLABSI in a pediatric oncology center. **Methods:** Since January 2024, ambulatory CLABSI events were identified by reviewing positive blood culture reports in an electronic surveillance system. The infection preventionist (IP) classified events as CLABSI or mucosal barrier injury (MBI) laboratory confirmed bloodstream infection (LCBI) using modified NHSN definitions. Ambulatory CVC-days were reported electronically for active patients with CVC and outpatient encounters. In March 2024, ACA investigation of patients admitted for ambulatory CLABSI was implemented using an electronic tool designed to address questions related to CVC care in the outpatient or home care setting. The ACA team consisted of an IP, outpatient nurse, and inpatient nurse. The team visited with the caregiver and/or patient for an interview and used this opportunity for just-in-time teaching to patient caregivers and staff. The results were entered into a database for lessons learned. **Results:** From January to November, there were 70 events classified as CLABSI in 67% and MBI-LCBI in 33%. Median (range) age was 5.0 (0.5-22.0) years. 39% had leukemia, 43% solid organ or brain tumor, and 14% were transplant recipients. 53% were neutropenic. Coagulase-negative staphylococcus was the most frequently isolated organism in 21% of patients followed by Escherichia coli in 17%. 47% had an in-person ACA interview; of which, 43% reported caregivers performing CVC care at home and 67% reported having been trained in the past 90 days. Table shows lessons learned from the ACA of the ambulatory CLABSI. **Conclusion:** ACA of ambulatory CLABSI is an important strategy to identify challenges of CVC care in the outpatient or home setting and to guide the design of a targeted improvement plan to reduce ambulatory.

**Figure:**